# Novel Pastes Containing Polymeric Nanoparticles for Dentin Hypersensitivity Treatment: An In Vitro Study

**DOI:** 10.3390/nano11113150

**Published:** 2021-11-22

**Authors:** Manuel Toledano-Osorio, Raquel Osorio, Estrella Osorio, Antonio L. Medina-Castillo, Manuel Toledano

**Affiliations:** 1 Faculty of Dentistry, Colegio Máximo de Cartuja s/n, University of Granada, 18071 Granada, Spain; mtoledano@correo.ugr.es (M.T.-O.); rosorio@ugr.es (R.O.); toledano@ugr.es (M.T.); 2 Analytic Chemistry Department, Faculty of Sciences, Campus Fuentenueva s/n, University of Granada, 18071 Granada, Spain; amedina@nanomyp.com; 3 NanoMyP, Spin-Off Company, Edificio BIC-Granada, Av. Innovación s/n, Armilla, 18016 Granada, Spain

**Keywords:** nanoparticles, zinc, dentin hypersensitivity, dentinal tubules, dentifrice

## Abstract

Tubule occlusion and remineralization are considered the two main goals of dentin hypersensitivity treatment. The objective is to assess the ability of dentifrices containing zinc-doped polymeric nanoparticles (NPs) to enduringly occlude the dentinal tubules, reinforcing dentin’s mechanical properties. Fifteen dentin surfaces were acid-treated for dentinal tubule exposure and brushed with (1) distilled water, or with experimental pastes containing (2) 1% of zinc-doped NPs, (3) 5% of zinc-doped NPs, (4) 10% of zinc-doped NPs or (5) Sensodyne^®^. Topographical and nanomechanical analyses were performed on treated dentin surfaces and after a citric acid challenge. ANOVA and Student–Newman–Keuls tests were used (*p* < 0.05). The main results indicate that all pastes produced tubule occlusion (100%) and reinforced mechanical properties of intertubular dentin (complex modulus was above 75 GPa). After the citric acid challenge, only those pastes containing zinc-doped NPs were able to maintain tubular occlusion, as specimens treated with Sensodyne^®^ have around 30% of tubules opened. Mechanical properties were maintained for dentin treated with Zn-doped NPs, but in the case of specimens treated with Sensodyne^®^, complex modulus values were reduced below 50 GPa. It may be concluded that zinc-doped NPs at the lowest tested concentration produced acid-resistant tubular occlusion and increased the mechanical properties of dentin.

## 1. Introduction

Regarding dental pathology, a change of paradigm has been witnessed. The prevalence of infectious dental diseases has decreased, mainly due to health-promotion strategies. In contrast, non-carious lesions have increased considerably, leading to Dental Hypersensitivity (DH), the prevalence of which has been reported to be between 4–74%, varying between different populations [1]. In Europe, the prevalence of non-carious lesions is particularly high in young adults (18–35 years), where 42% also suffer from DH [2].

DH is clinically described as a non-spontaneous, localized, intense pain of short duration that ceases when stimuli are removed [3]. Many theories have been proposed concerning the histopathological origin of DH; currently, the most accepted one is the “hydrodynamic theory” [4]. The theory states that the dentin hypersensitivity is characterized by a stimulus-induced fluid flow inside the dentinal tubules, followed by a nociceptor activation at the dentino-pulpal space [5]. This is why the dentinal tubules exposure to the oral cavity, caused by a loss of oral tissue (i.e., enamel, cementum or surrounding soft tissue) will lead to DH [6]. Therefore, this is the reason that tubule occlusion and dentin remineralization are to be considered the main objectives of DH treatment [7,8].

The use of bioactive materials, which are able to promote the deposit of calcium and phosphate on an inert scaffold, has been proposed to treat DH [8,9]. A bioactive glass (NovaMin^®^, NovaMin Technology Inc., Alachua, FL, USA) based on a 45S5 Bioglass^®^ (US Biomaterials Corp., Jacksonville, FL, USA) composition has been used in toothpaste formulations. The 45S5 are bioactive particles that set off an ionic reaction. At the oral cavity and after contact with saliva, the glass particles release calcium and phosphate ions, forming a calcium phosphate layer [8]. It has also been reported that these particles may have an abrasive effect on the dental surface and may produce a smear layer with the ability to close the tubules [8]. The durability of this layer is questioned [4,9,10,11], conferring a short-term level of tubule occlusion [12].

New biomaterials should facilitate sustained dentin remineralization and ensure a durable tubule occlusion [13,14,15]. Dentin infiltration using polymeric nanoparticles (NPs) that may work as calcium and phosphate sequestering materials has been proposed [14]. Anionic carboxylate sequences (COO-) on these NPs’ surfaces allow for complexation to cationic ions or molecules [14]. These NPs should be able to adhere to collagen and enable the formation of amorphous calcium and phosphate precursors for dentin remineralization [15]. There is clear evidence of the bioactivity of these NPs in apatite formation. They could be crucial for treating DH. The NPs may also be able to act as carriers of other biological factors for the enhancement of tissue mineralization. Zinc can also be quelated at these polymeric NPs, inhibiting collagen degradation and stimulating dentin remineralization [15,16].

The aim of this study was to assess the ability of dentifrices containing Zn-doped NPs to enduringly occlude the dentinal tubules, reinforcing dentin’s mechanical properties.

## 2. Materials and Methods

### 2.1. Nanoparticle Production and Description

PolymP-n active nanoparticles (NPs) (NanoMyP, Granada, Spain) were fabricated using a polymerization precipitation method [17]. The Flory–Huggins model (a thermodynamic approach) was used. It is based on the Hansen solubility parameters. The growth of polymeric chains was produced by a solvent vs. molecules interaction by hydrogen-bonding, dispersion and polar forces [17,18]. The NPs are composed of: (i) 2-hydroxyethyl methacrylate (backbone monomer), (ii) methacrylic acid as the functional monomer and (iii) ethylene glycol dimethacrylate (cross-linker). NPs are spherical and monodispersed; they were fabricated in a poor solvent, permitting the growth of polymeric chains. The phase-separation process was controlled by theoretical thermodynamic equations. The size of microspheres at full conversion was tuned by controlling the radius of the globules [18]. The complete synthesis of PolymP-n active nanoparticles is described by Medina-Castillo et al., (2010) [17] and Medina-Castillo (2020) [18]. For zinc-doping of the NPs (Zn-NPs), the NPs (30 mg) were immersed for 3 days under continuous shaking at room temperature in 15 mL aqueous solutions of ZnCl_2_ (zinc was at 40 ppm) (pH 6.5) in order to reach the adsorption equilibrium of a metal ion. The employed values came from the different zinc adsorption kinetic curves previously performed, through an inductively coupled plasma optical emission spectrometer [14]. Then, the suspension was centrifuged and the particles were separated from the supernatant. The attained ion complexation values were 2.15 ± 0.05 μg Zn/mg NPs [14]. Zinc was probed onto the NPs by an energy-dispersive analysis system and transmission electron microscopy [14]. The size of the NPs was assessed by hydrodynamic size distribution analysis through dynamic light scattering in deionized water. The NPs’ size did not change after zinc-doping. The attained average size and standard deviation of Zn-NPs was 225.93 ± 8.88 nm [15].

### 2.2. Experimental Toothpaste Preparation

Three different toothpastes were prepared containing 1, 5 or 10 wt% Zn-doped NPs. None of these toothpastes included any ingredient with remineralizing or abrasive capacities.

### 2.3. Dentin Disks Preparation

Fifteen non-carious human third molars were obtained with previously informed consent from donors (18–25 years of age), under a research protocol approved by the Institution Review Board of the University of Granada, Spain (405/CEIH/2017). The teeth were stored in a thymol solution (0.01% *w*/*v*) at 4 °C. Fifteen dentin discs (0.75 mm ± 0.08 mm thick) were automatically sectioned (Isomet 4000, Buehler, Lake Bluff, IL, USA) at the mid-coronal portion of the tooth. Just one slice was obtained from each molar. The surfaces were polished with silicon carbide grinding papers from 800 up to 4000 grit, followed by final polishing steps performed with diamond pastes from 1 μm up to 0.25 μm (Struers LaboPol-4; Struers GmbH, Hannover, Germany). The demineralization challenge was performed following the method described by Osorio et al. [6]. Dentinal tubules were exposed by immersing the disks into a 0.5 M solution of ethylenediaminetetraacetic acid (EDTA) (pH 7.4) for 2 min [9] and then rinsing them with deionized water for 30 s. The disks were brushed with the experimental toothpastes for 2 min each. Five experimental groups, including three specimens in each one, were tested: (1) toothpaste containing 1% of Zn-NPs (P1%), (2) containing 5% of Zn-NPs (P5%), (3) 10% of Zn-NPs (P10%) and (4) Sensodyne^®^ Repair and Protect (with NovaMin^®^ technology) as positive control; (5) discs brushed with distilled water were also analyzed. An electric toothbrush (Oral-B Vitality Precision Clean, P&G Barcelona, Spain) was utilized for the 2-min brushing procedure. The brush was used and stabilized with the aid of a micro-manipulator (World Precision Instruments Ltd., London, UK); a force of 70 g was applied in order to ensure a uniform and constant pressure. For each group, a different and new soft brush was assigned. Then, specimens were rinsed with deionized water (60 s) and immersed in artificial saliva for 24 h (50 mM/L HEPES, 5 mM/L CaCl_2_·2H_2_O, 0.001 mM/L ZnCl_2_, 150 mM/L NaCl (pH 7.2)) to avoid bacteria. A total of 100 U/mL penicillin and 1000 μg/mL streptomycin were also added to the solutions. The discs were sectioned into two halves. The first halves were submitted to the topography and nanomechanical properties evaluation, and finally, scanning electron microscopy was performed on the same specimens. The other halves were subjected to an acid challenge. For this purpose, specimens were immersed in a citric acid solution (6 wt%) (pH 1.5) for 1 min and rinsed with deionized water for 30 s [9]. These dentin disc halves were then submitted to the same range of tests as the first ones.

### 2.4. Atomic Force Microscopy (AFM) Imaging

The imaging procedure was performed in the tapping mode, by means of AFM (Nanoscope V, Digital Instruments, Veeco Metrology group, Santa Barbara, CA, USA) using a calibrated vertical-engaged piezo-scanner. A silicon nitride tip (10 nm in radius) was attached to the end of an oscillating cantilever; it came into intermittent contact with the dentin surface at the lowest point of the oscillation. Changes in the vertical position of the AFM tip (resonance frequencies of approximately 330 kHz) provided the height of the dentin images, registered as bright and dark regions. Measurements were performed under hydrated conditions using a wet cell. Three randomized regions of approximately 15 μm × 15 μm were obtained from each surface, with a slow scan rate (0.1 Hz).

### 2.5. Nanomechanical Properties Analysis

A nanomechanical properties analysis of the dentin surfaces was performed using a Hysitron Ti Premier nanoindenter (Hysitron, Inc., Minneapolis, MN, USA), provided with a commercial nano-dynamic mechanical analysis (nano-DMA) package. The nanoindenter tip was calibrated. Calibration was executed against a fused quartz sample. A quasistatic force setpoint of 2 μN was employed in order to maintain contact between the tip and the sample surface. A dynamic (oscillatory) force of 2 μN was superimposed on the quasistatic signal at a frequency of 100 Hz. Based on a value of 69.6 GPa and a calibration-reduced modulus for the fused quartz, the best-fit spherical radius for the tip was found to be approximately 85 nm, taking into account the selected nano-DMA scanning parameters. Modulus mapping of our samples was also performed by imposing a quasistatic force setpoint (Fq = 2 μN) to which a sinusoidal force of amplitude FA = 0.10 μN and frequency f = 100 Hz was superimposed. The resulting deformation (displacement) at the indentation site was monitored as a function of time. Dentin specimens were scanned under a hydrated condition. To maintain the hydration of the dentin samples while eliminating problems related to the meniscus forces transferred from droplets of fluid to the indenter [19], a drop (1.5 mL) of 99.4% ethylene glycol was applied to the polished sample surface [20].

Three randomized regions, approximately 15 μm × 15 μm in size, were created at each surface using a scanning frequency of 0.2 Hz. Ten complex modulus data were collected from each of these scanned regions at the intertubular and intratubular dentin zones. For those images in which tubules were not easily identified, intratubular dentin values were acquired after locating tubules at the corresponding topographical image, displayed by the nanoindenter for each mapping. Under steady conditions, with the application of a quasistatic force, the indentation modulus of the tested dentin samples, E, was obtained by the application of different models, which relate the indentation depth, D, and force, F [21].

### 2.6. Field Emission Scanning Electron Microscopy (FESEM)

After AFM and nano-DMA analyses, specimens were fixed by means of a 2.5% glutaraldehyde solution in 0.1 mol/L sodium cacodylate buffer for 24 h. Samples were then subjected to critical-point drying (Leica EM CPD 300, Wien, Austria), and finally, they were sputter-coated with carbon using a sputter-coating Nanotech Polaron-SEMPREP2 (Polaron Equipment Ltd., Watford, UK). Specimens were observed with a field emission scanning electron microscope (FESEM Gemini, Carl Zeiss, Oberkochen, Germany); the employed accelerating voltage was 3 kV. To analyze for tubule occlusion, three 2500× magnification images were taken at randomized areas for each specimen. Two examiners were previously calibrated on FESEM raw images and definitions of: (1) open (O), when the tubules were completely empty, (2) partially filled (P), when the tubules were not completely occluded but had some material inside and (3) filled (F), when tubules were completely occluded [22]. A blind testing was performed; examiners were unaware of the study groups. The average of the assessments was calculated for each specimen. The percentage of total open tubules (%Total) was calculated by dividing the mean open tubules found in each group (Group OT) by the total number of not-occluded tubules in the control images (Control OT): %Total = (Group OT/Control OT) ×100. Some other images at a higher magnification were taken to ascertain the interactions between dentin and particles.

### 2.7. Statistical Analysis

Normal data distribution was determined by Kolmogorov–Smirnov (*p* > 0.05). Multiple ANOVA and Student–Newman–Keuls comparison tests were used to compare the several experimental paste groups (*p* < 0.05). The IBM SPSS Statistics 24 computer software (accessed on 30/06/2020) was employed.

## 3. Results

### 3.1. AFM Imaging

**Figure 1:**
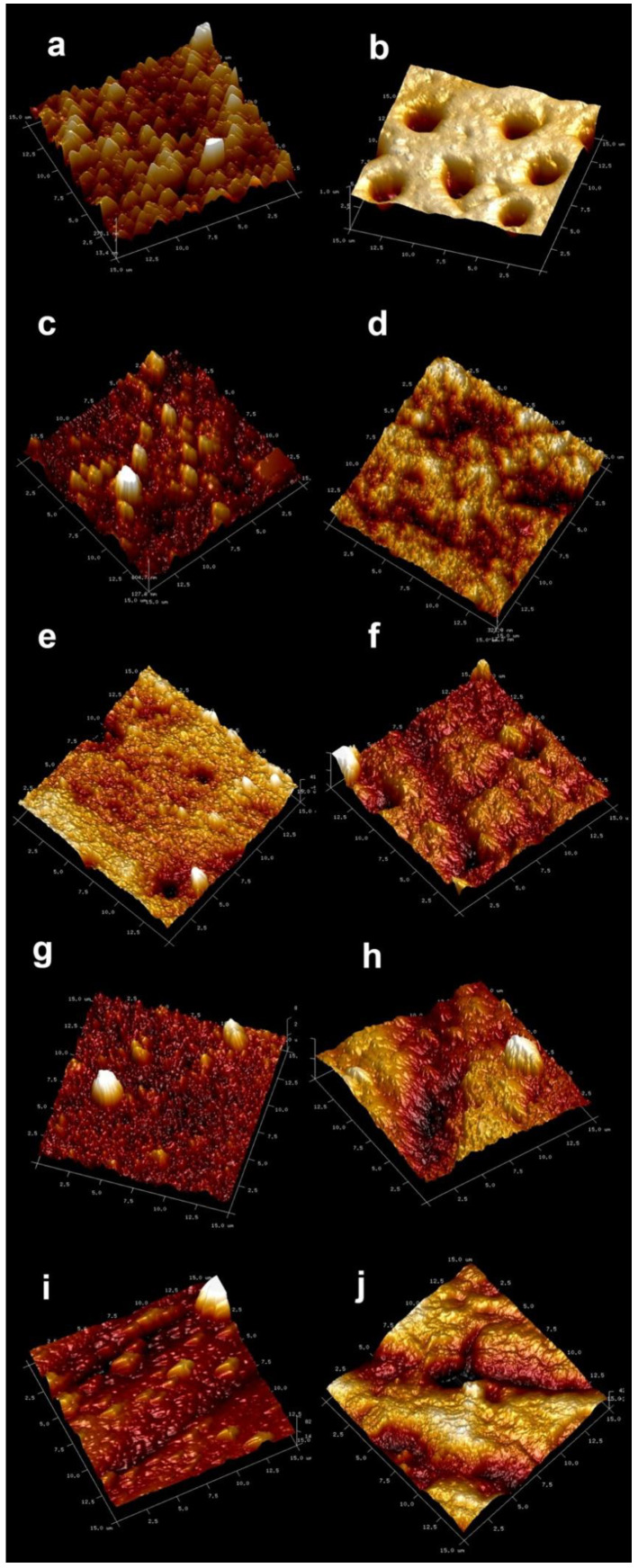
AFM images of the dentin surfaces after EDTA treatment and: (**a**) being brushed with distilled water and (**b**) after-treated with citric acid; (**c**) being brushed with toothpaste containing 1% of Zn-NPs and (**d**) after-treated with citric acid; (**e**) being brushed with toothpaste containing 5% of Zn-NPs and (**f**) after-treated with citric acid; (**g**) being brushed with toothpaste containing 10% of Zn-NPs and (**h**) after-treated with citric acid; and (**i**) being brushed with Sensodyne^®^ toothpaste and (**j**) after-treated with citric acid. Scan size is 15 μm × 15 μm.

The AFM images of treated dentin specimens are shown in Figure 1. Morphological differences may be encountered between groups. After P1%, P5% or P10% treatments (Figure 1c,e,g), the groups presented complete tubular occlusion. It was not possible to observe collagen fibrils or dentinal tubules, as the surfaces appeared covered by a continuous and homogeneous layer. Some crystal growth was evidenced. After the citric acid (CA) challenge, dentin tubules were partially or totally occluded in all these groups (P1%+CA, P5%+CA and P10%+CA are shown in Figure 1d,f,h). After submitting the samples to the citric acid challenge, specimens brushed with distilled water (Figure 1b) showed open tubules.

### 3.2. Nanomechanical Properties Analysis

**Figure 2:**
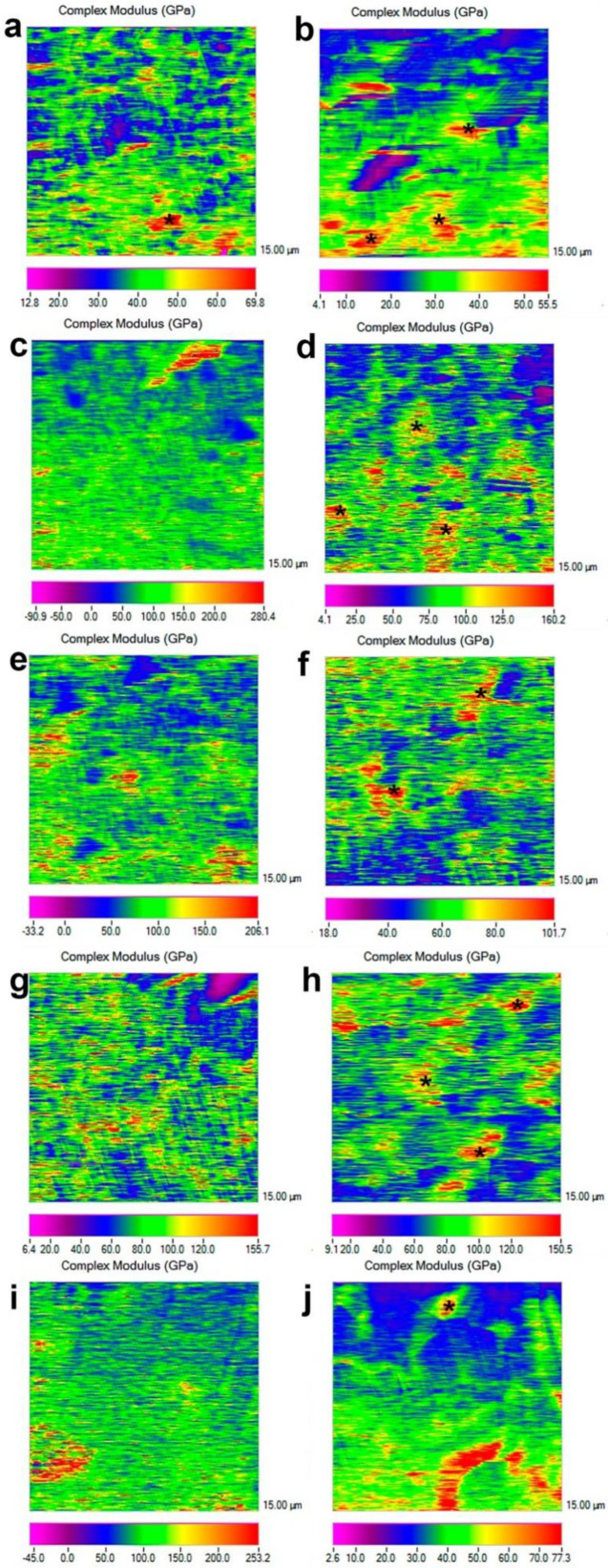
Nano-DMA mapping of the EDTA-treated dentin surfaces, after the following the treatments: (**a**) brushed with distilled water and (**b**) after-treated with citric acid; (**c**) brushed with toothpaste containing 1% of Zn-NPs and (**d**) after-treated with citric acid; (**e**) brushed with toothpaste containing 5% of Zn-NPs and (**f**) after-treated with citric acid; (**g**) brushed with toothpaste containing 10% of Zn-NPs and (**h**) after treated with citric acid; and (**i**) brushed with Sensodyne^®^ toothpaste and (**j**) after treated with citric acid. The pixel data array for the mapping is organized according to the complex modulus distribution that concurs with a clear delimitation between intertubular and peritubular dentin. For the color scheme shown, the red color corresponds to the highest value of the locally measured complex modulus (E*), likely corresponding to the highest resistance to deformation and potentially associated with mineral precipitation. In most of the cases, it corresponds to intratubular locations (asterisks). Scale bars are in GPa. Scan size is 15 μm × 15 μm.

The complex modulus mapping of dentin surfaces brushed with the experimental pastes are presented in Figure 2. Means and standard deviations are displayed in Table 1. In general, after storage in artificial saliva for 24 h, the dentin specimens brushed with any of the tested pastes had augmented complex modulus values for intratubular (a two- to three-fold increase) and intertubular (a two-fold increase) dentin (Table 1). After the citric acid challenge, specimens brushed with pastes containing Zn-doped NPs maintained complex modulus values above those specimens that were brushed with distilled water and even greater values than those obtained by the samples treated with Sensodyne^®^ (Table 1). The complex modulus of specimens brushed with Sensodyne^®^ and submitted to the citric acid challenge (intratubular mean: 51.10, SD: 13.84 and intertubular mean: 48.41, SD: 8.83) attained similar values to the specimens that had been treated with EDTA and brushed with distilled water for both intratubular (mean: 57.37, SD: 6.82) and intertubular dentin (mean: 45.55, SD: 6.70).

### 3.3. Field Emission Scanning Electron Microscopy (FESEM)

Tubular occlusion assessments for the different dentin surfaces are expressed in Table 2. The number of open, partially filled or filled observed tubules is displayed. The percentage of total open tubules (%Total) was measured by dividing the mean of open tubules for each group by the total number of tubules shown in the images of control specimens (EDTA+DW and EDTA+DW+CA, respectively).

**Figure 3:**
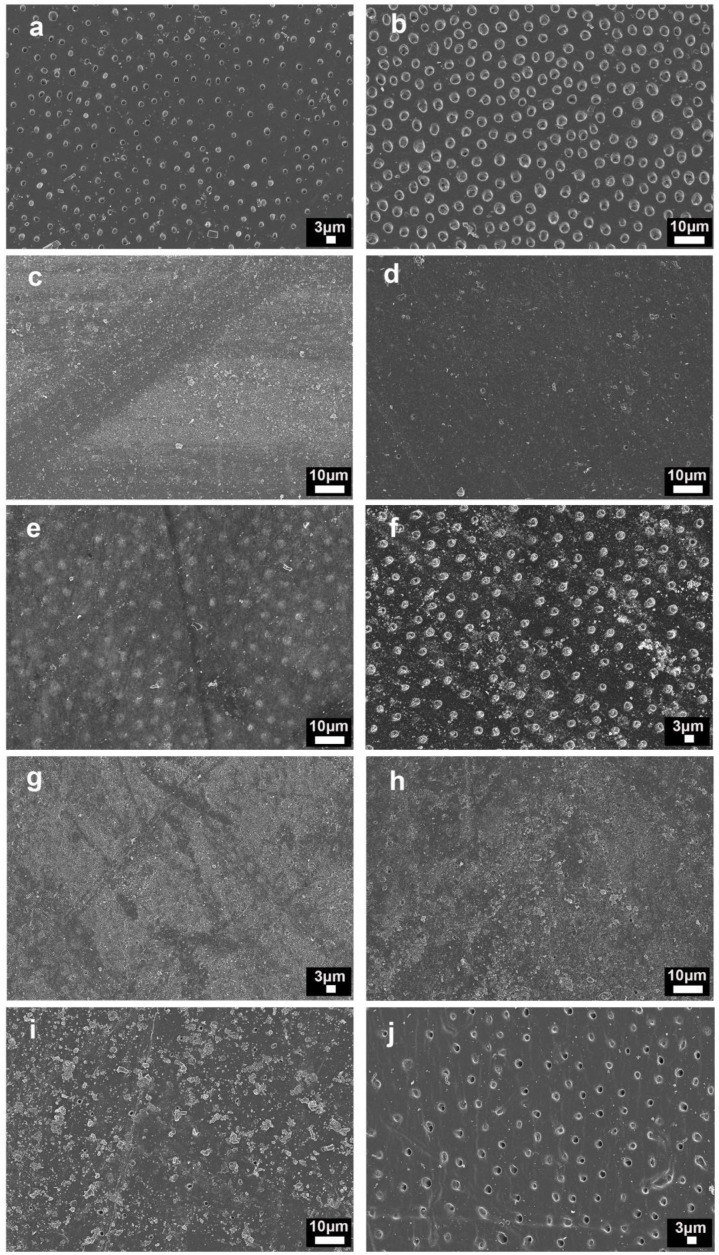
FESEM images of the dentin surfaces after EDTA-treatment and: (**a**) brushed with distilled water and (**b**) after-treated with citric acid; (**c**) brushed with toothpaste containing 1% of Zn-NPs and (**d**) after-treated with citric acid; (**e**) brushed with toothpaste containing 5% of Zn-NPs and (**f**) after-treated with citric acid; (**g**) brushed with toothpaste containing 10% of Zn-NPs, and (**h**) after-treated with citric acid; and (**i**) brushed with Sensodyne^®^ toothpaste and (**j**) after-treated with citric acid. All images are taken at 2500× magnification.

Representative FESEM images are displayed in Figure 3. All tested pastes effectively occluded 100% of the dentinal tubules after just one application (Figure 3c,e,g,i). After the citric acid challenge, dentin surfaces treated with pastes containing Zn-NPs remained with most of the tubules occluded (Figure 3d,f,h). In the case of Sensodyne^®^ toothpaste, 30% of tubules were found to be open after FESEM examination (Figure 3j).

**Figure 4:**
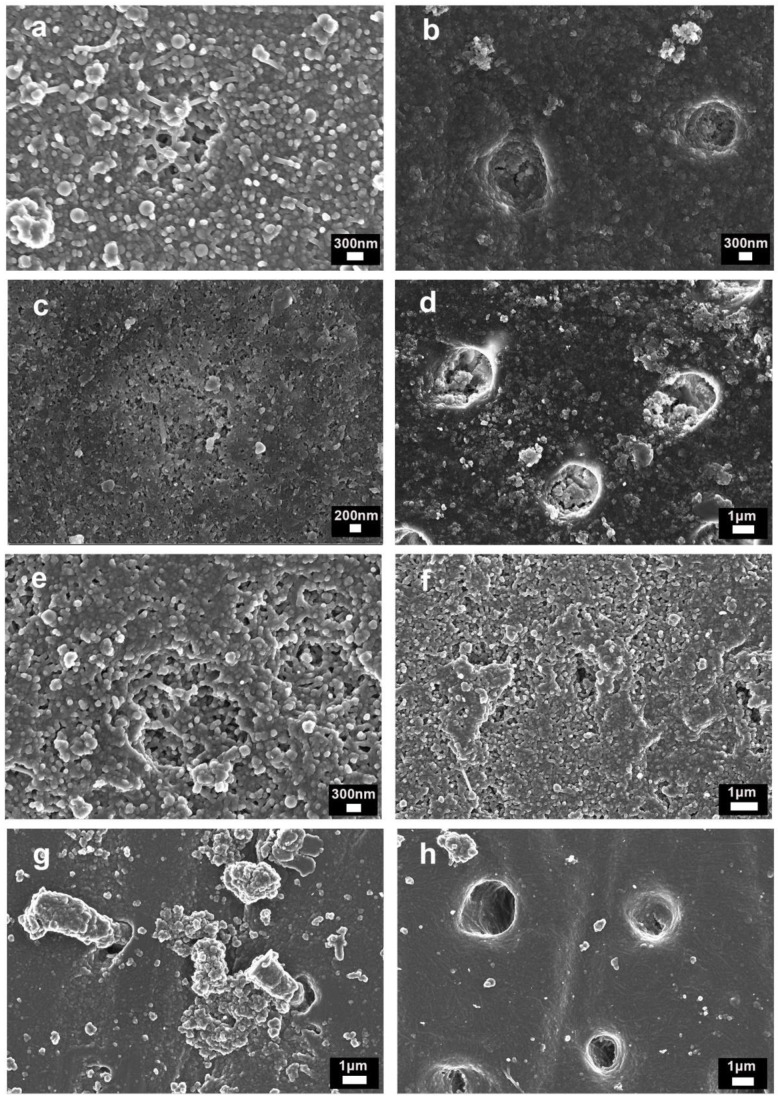
FESEM images of the dentin surfaces after EDTA-treatment and: (**a**) brushed with toothpaste containing 1% of Zn-NPs, where a single tubule occluded by NPs and new mineral formations were visible; (**b**) brushed with toothpaste containing 1% of Zn-NPs, for which occluded tubules were observed after acid application. Intertubular dentin was also covered by NPs and new minerals; (**c**) brushed with toothpaste containing 5% of Zn-NPs. A dentinal tubule occluded by NPs and new mineral formations were detected; (**d**) brushed with toothpaste containing 5% of Zn-NPs and after-treated with citric acid. Occluded and partially filled tubules are shown. Intertubular dentin was remineralized; (**e**) brushed with toothpaste containing 10% of Zn-NPs. Filled tubules and intertubular dentin covered by NPs and new minerals; (**f**) brushed with toothpaste containing 10% of Zn-NPs and after-treated with citric acid. A tiny tubule (about 250 nm) orifice was detectable on the dentin surface; (**g**) brushed with Sensodyne^®^ toothpaste, where tubules were occluded by smear plugs, which were dislodged from the main dentinal orifice; (**h**) brushed with Sensodyne^®^ toothpaste and after-treated with citric acid, for which intertubular dentin was demineralized and collagen fibers were observable.

At a higher magnification, filled tubules and intertubular dentin covered by NPs and new minerals are observable after the application of Zn-NP-containing pastes. In some cases, after the citric acid application, the tubules’ orifices were observable but the tubular entrances remained occluded (Figure 4a–e). Occasionally, after the Sensodyne^®^ application, the tubules appeared occluded by smear plugs, which were dislodged from the main dentinal orifice (Figure 4g). After citric acid application for the Sendodyne^®^-treated dentin, tubules were not only opened, but intertubular dentin was also demineralized, becoming observable some collagen fibers (Figure 4h).

## 4. Discussion

Even when DH is a prevalent pathology, there is not a successful treatment that may help to reduce long-term pain. There is some uncertainty regarding the exact pain mechanism in DH, but from a clinical perspective, therapies for DH should interact with the hydrodynamic sequence either within the neural transmission pathway at the pulp/dentin space or at the surface of the patent dentin tubules [23]. It is clear that it is necessary to reduce the dentinal tubules’ opening on a permanent basis, as material deposition and occluding tubules correlates with DH symptom-relief [24].

For the present research, all tested pastes are able to create deposits and to produce occluding tubules with just one application (Figure 1 and Figure 3). The use of several desensitizing toothpastes containing calcium sodium phosphosilicate, strontium acetate, arginine calcium carbonate or stannous fluoride occlude the openings of the dentinal tubules [24]. All these dentifrices create a smear-layer-like coating able to produce tubule plugs [1].

Two main shortcomings are associated with occluding tubules through this formed smear-layer coating. The first one is the vulnerability to its subsequent dissolution by acids or saliva, as well as being worn away by further tooth-brushing or other abrasive substances [9]. To demonstrate the tubules’ occlusion resistance, an acid challenge is usually performed on treated surfaces [6,9]. In this experiment, only the pastes containing polymeric zinc-doped NPs were able to produce an occluding tubule resistant to citric acid. After citric acid application, for Sensodyne^®^ treated specimens, about 30% of the tubules were newly opened (Table 2), showing that Sensodyne^®^, as with other pastes containing inorganic particles/crystals, acts in part by creating a dentin smear-layer through dentinal abrasion. How the formed smear plugs on Sensodyne^®^-treated dentin are easily dislodged from tubules (Figure 4g) may be observed by FESEM. In the case of zinc-doped NPs, it is not possible to produce dentin abrasion due to the very low surface hardness of polymeric NPs compared to the dentin or enamel values. The second problem is the produced abrasion on the dentin structure [1]. These toothpastes accomplished the ISO specification, not exceeding an RDA of 250, which is considered the safe limit for hard tissues. However, it should also be considered that tooth wear is multifactorial, and toothpaste abrasives may play a small role in DH development if compared to other contributing factors [1]. Sensodyne^®^ contains particles which have a higher hardness value than dentin, producing abrasion. In terms of abrasive wear during tooth-brushing, it would be desirable to have particles with lower hardness values than dentin, in order to minimize the loss of the natural structure [25]. The crucial role of erosion in contributing to dentinal sensitivity has been previously recognized [26] and a causal relationship has been demonstrated by the examination of a large dataset of patients with severe erosive tooth wear [27].

It has to be stated that when applying Sensodyne^®^, tubular occlusion may also be achieved due to some of the paste’s constituents, which are supposed to create crystals inside the tubules upon hydration [9]. It can be observed that, after Sensodyne^®^ application, intratubular dentin nanohardness attained high mean values (about 140 GPa, Table 1), a fact that could be attributed to these previously mentioned crystals [28]. However, these formed crystals, after Sensodyne^®^ application, do not appear to be completely acid-resistant [9] since intratubular nanohardness values were reduced to 50 GPa after citric acid application (Table 1).

Other commercially available toothpastes different from Sensodyne^®^ have been formulated with bioactive compounds, such as amorphous silica and/or hydroxyapatite, for the treatment of DH, and have also shown the ability to produce mineral deposits on the dentin surface that lead to the occlusion of dentinal tubules [29]. However, the real abrasive effect and durability of these deposits remains to be ascertained.

After applying any of the pastes with the experimental NPs, tubules were completely occluded and were non-detectable in AFM or FESEM images (Figure 1, Figure 3 and Figure 4). NPs preserve their previously shown ability to be adsorbed on the dentin and are able to quelate calcium and phosphate [16]. It is important to note that it was demonstrated that the toothpaste components did not encapsulate the NPs, impede the NPs’ deposition on dentin nor reduce their quelating ability. Therefore, the possible interaction between the different ions and surfactant composing the toothpaste was discarded. Moreover, it is also shown in the present research that the beneficial effect of Zn-NPs on dentin does not require the high NPs concentration (10%) that was tested in advance [15]. A total of 1 wt% of NPs was enough to produce the desired effects of occluding tubules and the reinforcement of dentin’s mechanical properties. It seems that NPs can be lodged into the dentinal tubules during brushing procedures due to their small size (Figure 4a–e). Only a few tubules remained open and with maximum narrowing of the tubular lumen (Figure 4f). The percentage of total open tubules after applying the citric acid and the Zn-NP pastes was nearly zero (Table 2). Moreover, intratubular dentin nanohardness was above 110 GPa after applying Zn-NP pastes and remained above 80 GPa after the citric acid challenge (Table 1).

Attempts to inhibit dentin demineralization with zinc oxide have previously been performed with success. A total of 1% of zinc oxide microparticles included in toothpastes was found to have an inhibitory effect on dentine demineralization, being effective in the prevention of root caries [30]. This may be considered an additional benefit of using the presented pastes containing Zn-NPs when treating DH.

Clinical studies are needed to prove the expected relief of DH symptoms when using the tested Zn-doped NP pastes, which may effectively occlude dentinal tubules. These pastes may be extremely beneficial, as they should not only provide clinical satisfactory outcomes for pain reduction, but also produce a mechanical reinforcement of the treated dentin surface. It may be considered a big advantage for these patients, who are usually prone to the continuation of erosive lesions [1]. As stated in Table 1, complex modulus values attained at the intertubular dentin after applying Zn-NP pastes are above 70 GPa, and these values remained unchanged after acid application, confirming a previously encountered remineralization effect of these Zn-NP for demineralized dentin [16,31]. However, when analyzing the complex modulus obtained for intertubular dentin after Sensodyne^®^ application, a high complex modulus of 90 GPa is evidenced, probably due to crystal formation (Figure 1i). However, after acid application, these values were lowered to 50 GPa, stating a lack of durability of the produced effect when using Sensodyne^®^ paste. It is then supposed that patients who discontinue the use of Sensodyne^®^ will promptly notice the absence of the previously related clinical benefits.

Ideally, sustained zinc liberation is expected to occur from the Zn-NPs [32], and it may also produce other beneficial effects, such as metalloproteinases inhibition [33] (as they are implicated in erosive cervical lesions [6]) or even periodontal [34] and cariogenic [35] biofilm formation inhibition. The incorporation of ZnO or other different zinc salts in toothpastes or mouthwashes have been shown to enable the fighting of gingivitis and even periodontitis, due to their bactericidal and anti-inflammatory effects [36].

Even when the present in vitro results are promising, it is clear that clinical studies will be required to demonstrate that they can be converted into benefits for patients.

## 5. Conclusions

It can be stated that these toothpastes with Zn-doped NPs could pose as a potential treatment for DH. These toothpastes have been shown in vitro, not only to occlude dentinal tubules, but also to enhance the mechanical properties of the remaining dental structure, therefore avoiding the loss of hard tissue produced by other toothpastes for non-carious cervical lesions. However, they should be considered as a treatment for patients suffering from DH only after performing clinical experimental studies.

## Figures and Tables

**Table 1:** Means and standard deviations (SD) of the complex modulus (GPa) of dentin at intertubular and intratubular spaces, after the different treatments.

Dentin Treatment	Complex Modulus (GPa)			
	Intratubular Dentin		Intertubular Dentin	
	Mean	SD	Mean	SD
Distilled water (DW)	57.37 b	6.82	45.55 B	6.70
Zn-NP paste P1%	147.87 f	19.94	87.82 D	10.50
Zn-NP paste P5%	133.99 def	14.15	73.15 C	8.04
Zn-NP paste P10%	110.64 d	6.97	79.31 CD	3.39
Sensodyne paste ®	140.93 ef	39.12	89.26 D	12.86
DW + Citric Acid (CA)	24.18 a	4.21	31.93 A	6.15
Zn-NP paste P1% + CA	116.75 de	6.52	74.43 C	6.59
Zn-NP paste P5% + CA	83.28 c	11.15	69.72 C	8.26
Zn-NP paste P10% + CA	127.38 def	13.21	77.54 CD	7.73
Sensodyne^®^ paste + CA	51.10 b	13.84	48.41 B	8.83

ANOVA Results: Intratubular dentin F = 39.65; *p* < 0.001; Intertubular dentin F = 32.39; *p* < 0.001. Groups with distinct letters within each dentin type are significantly different after multiple Student–Newman–Keuls comparisons (*p* < 0.05).

**Table 2:** Tubular occlusion assessment for the several treated dentin surfaces. The number of open, partially filled or filled observed tubules is displayed. The percentage of total open tubules (%Total) was measured by dividing the mean of open tubules for each group by the total number of tubules shown in the EDTA or EDTA+CA control images ((open tubules)/total number of control images × 100).

Dentin Treatment	Tubular Occlusion		
	Open Tubules	Filled/Partially Filled Tubules	%Total Open Tubules
EDTA	273	0	100
EDTA+Zn-NP paste P1%	0	0	0
EDTA+Zn-NP paste P5%	0	3	0
EDTA+Zn-NP paste P10%	0	9	0
EDTA+Sensodyne^®^ paste	0	25	0
EDTA+Citric Acid (CA)	274	0	100
EDTA+Zn-NP paste P1% + CA	0	12	0
EDTA+Zn-NP paste P5% + CA	2	189	0.007
EDTA+Zn-NP paste P10% + CA	3	9	0.01
EDTA+Sensodyne^®^ paste +CA	86	30	31.38

## Data Availability

The data presented in this study are available on request from the corresponding author. The data are not publicly available due to general data protection restrictions.
